# Can CanAssist Breast guide the duration of extended endocrine therapy?

**DOI:** 10.1016/j.breast.2026.104950

**Published:** 2026-10-29

**Authors:** Yuewei Song, Pengfei Jia, Min Jiang

**Affiliations:** a Beijing University of Chinese Medicine, Beijing, China; b Department of Oncology, Dongfang Hospital, Beijing University of Chinese Medicine, Beijing, 100078, China

Durgekar et al. reported 10-year distant recurrence-free survival (DRFS) from randomization of 84.1% versus 74.3% among 79 CanAssist Breast (CAB) ultra-high-risk (UHR) patients assigned 5 versus 2.5 additional years of letrozole in IDEAL [1], [2]. They suggest benefit only for UHR patients, potentially sparing the remaining 88% from overtreatment. The clinical question is whether low- or high-risk CAB results justify omitting the final 2.5 years.

No treatment-by-CAB interaction was reported. From published hazard ratios (HRs) and 95% CIs, we estimated log-HR standard errors and calculated a fixed-effect inverse-variance summary HR of 0.99 (95% CI, 0.58–1.69) for the low- and high-risk categories combined. The UHR-to-non-UHR ratio of HRs was 0.60 (95% CI, 0.19–1.83; P ≈ 0.37) [1], [3]. These approximate results do not exclude heterogeneity, but neither establish greater relative benefit in UHR nor rule out meaningful benefit elsewhere.

Patient-level interaction testing, category-by-arm event counts, and 95% CIs for absolute 10-year DRFS differences could clarify how much benefit might be forgone by shortening therapy and whether clinically meaningful benefit remains plausible outside UHR. This would inform the trade-off between recurrence reduction and toxicity; CAB low- or high-risk status alone cannot establish that continued therapy lacks meaningful benefit.

## CRediT authorship contribution statement

**Yuewei Song:** Conceptualization, Investigation, Writing – original draft, Writing – review & editing. **Pengfei Jia:** Conceptualization, Writing – review & editing. **Min Jiang:** Conceptualization, Writing – review & editing.

## Data availability

No new datasets were generated. All source data analyzed in this Correspondence are available in the cited publications.

## Ethics approval and consent

Not applicable.

## Funding

None.

## Declaration of competing interests

Yuewei Song, Pengfei Jia, and Min Jiang declare no conflicts of interest.
